# Outdoor air pollution, subtypes and severity of ischemic stroke – a small-area level ecological study

**DOI:** 10.1186/1476-072X-13-23

**Published:** 2014-06-17

**Authors:** Ravi Maheswaran, Tim Pearson, Sean D Beevers, Michael J Campbell, Charles D Wolfe

**Affiliations:** 1 Public Health GIS Unit, School of Health and Related Research, University of Sheffield, Regent Court, 30 Regent Street, Sheffield S1 4DA, UK; 2 Environmental Research Group, King’s College London, 150 Stamford Street, London SE1 9NH, UK; 3 Design, Trials and Statistics Section, School of Health and Related Research, University of Sheffield, Regent Court, 30 Regent Street, Sheffield S1 4DA, UK; 4 Division of Health and Social Care Research, King’s College London and National Institute for Health Research Biomedical Research Centre, Guy’s and St Thomas’ NHS Foundation Trust and King’s College London, 7th Floor, Capital House, 42 Weston Street, London SE1 3QD, UK

**Keywords:** Stroke, Air pollution, Environmental exposure, Subtypes, Severity

## Abstract

**Background:**

Evidence linking outdoor air pollution and incidence of ischemic stroke subtypes and severity is limited. We examined associations between outdoor PM_10_ and NO_2_ concentrations modeled at a fine spatial resolution and etiological and clinical ischemic stroke subtypes and severity of ischemic stroke.

**Methods:**

We used a small-area level ecological study design and a stroke register set up to capture all incident cases of first ever stroke (1995–2007) occurring in a defined geographical area in South London (948 census output areas; population of 267839). Modeled PM_10_ and NO_2_ concentrations were available at a very fine spatial scale (20 meter by 20 meter grid point resolution) and were aggregated to output area level using postcode population weighted averages. Ischemic stroke was classified using the Oxford clinical classification, the Trial of Org 10172 in Acute Stroke Treatment (TOAST) etiological classification, National Institutes of Health Stroke Scale (NIHSS) score and a pragmatic clinical severity classification based on Glasgow coma score, ability to swallow, urinary continence and death <2 days of stroke onset.

**Results:**

Mean (SD) concentrations were 25.1 (1.2) ug/m^3^ (range 23.3-36.4) for PM_10_ and 41.4 (3.0) ug/m^3^ (range 35.4-68.0) for NO_2_. There were 2492 incident cases of ischemic stroke. We found no evidence of association between these pollutants and the incidence of ischemic stroke subtypes classified using the Oxford and TOAST classifications. We found no significant association with stroke severity using NIHSS severity categories. However, we found that outdoor concentrations of both PM_10_ and NO_2_ appeared to be associated with increased incidence of mild but not severe ischemic stroke, classified using the pragmatic clinical severity classification. For mild ischemic stroke, the rate ratio in the highest PM_10_ category by tertile was 1.20 (1.05-1.38) relative to the lowest category. The rate ratio in the highest NO_2_ category was 1.22 (1.06-1.40) relative to the lowest category.

**Conclusions:**

We found no evidence of association between outdoor PM_10_ and NO_2_ concentrations and ischemic stroke subtypes but there was a suggestion that living in areas with elevated outdoor PM_10_ and NO_2_ concentrations might be associated with increased incidence of mild, but not severe, ischemic stroke.

## Background

Stroke is a common cause of mortality and morbidity worldwide [1]. The potential importance of outdoor air pollution as a risk factor for stroke is being increasingly recognized [2]. Studies suggest that associations are stronger for ischemic than for hemorrhagic stroke [3,4]. Conceptually, short-term spikes in pollution levels may acutely trigger a stroke, for example through plaque rupture or transient increases in blood coagulability, whilst chronic exposure may exert a chronic effect through acceleration of atherosclerosis [5]. These potential mechanisms for air pollution mediated cerebrovascular disease primarily relate to ischemic stroke.

Attention is turning to investigation of associations between air pollutants and subtypes of ischemic stroke as these have different pathophysiological mechanisms. The associations have to date been examined in four studies investigating acute exposure effects [6-9]. Three found associations with stroke due to large vessel disease [6-8] while three found associations with stroke due to small vessel disease or lacunar stroke [7-9]. Chronic exposure effects of outdoor air pollutants on incidence of ischemic stroke subtypes have, however, not been examined.

Investigation of chronic exposure effects would ideally use cohort studies but these could be expensive as large cohorts may be needed to obtain adequate power. Ecological studies offer an alternative study design. Traditional ecological studies have well-recognized limitations, particularly ecological bias. Small-area level ecological studies address many of these limitations as populations tend to be relatively more homogenous within small geographical areas with regard to socioeconomic characteristics and environmental exposures [10]. In addition, this study design can capture spatial variation in road traffic related pollution at a fine spatial scale [11]. This is useful as traffic related pollution levels can vary substantially within short distances of main roads. We have previously used the small-area level ecological study design to investigate associations between air pollution and stroke [4,12,13].

We previously observed that air pollutants are more strongly associated with stroke mortality than with hospital admissions for stroke [13]. One potential explanation is that air pollution is more likely to cause severe stroke resulting in death. However, others examining acute effects have reported associations with mild but not severe stroke and the authors used mild stroke as a proxy for stroke caused by small vessel disease [14].

The aim of our study was to investigate the associations between outdoor air pollution concentrations and the incidence of ischemic stroke subtypes and severity [15]. We used a small area level ecological study design and examined the effects of pollutants on etiological and clinical ischemic stroke subtypes as well as on the incidence of mild and severe ischemic stroke.

## Methods

### Stroke incidence data

Stroke incidence data were obtained from the South London Stroke Register, a population-based register set up in 1995 and designed to capture all incident cases of first ever stroke occurring amongst the resident population living in a defined geographical area of south London [16]. The area was expanded in 2004 but for this study, we only included the part that was consistently in the Register area from 1995–2007. The Register used multiple sources of information to capture incident cases of stroke. Hospital and community notification sources included accident and emergency records, hospital staff, brain imaging requests, death certificates, coroners’ records, general practitioners, community nurses and therapists, bereavement officers, social services, hospital based stroke registries, general practice computer records and notification by patients or relatives. Estimated completeness of case capture was 80-88% [17,18]. The study had approval from the ethics committee of Guy’s and St Thomas’ Hospital Trust, King’s College Hospital.

All patients were examined within 48 hours of notification and investigated using a standardized protocol which included neuroimaging, with additional investigation for ischemic stroke using an investigation algorithm incorporating carotid duplex and transcranial Doppler scanning, trans-thoracic echocardiography, trans-esophageal echocardiography and hematological investigation as appropriate [19]. The Oxford clinical classification was implemented in 1995 when the Register commenced, with cerebral infarction being categorized as total anterior circulation infarct (TACI), partial anterior circulation infarct (PACI), posterior circulation infarct (POCI) and lacunar infarct (LACI) [20]. The Trial of Org 10172 in Acute Stroke Treatment (TOAST) classification of ischemic stroke subtypes based on etiology was fully implemented from 2000 [21]. We examined three TOAST categories – large artery atherosclerosis, cardioembolism and small vessel occlusion. Both classification systems have been used in previous studies examining the acute effects of air pollution exposure on ischemic stroke subtypes [6-9].

The National Institutes of Health Stroke Scale (NIHSS) was fully implemented in 2001 and an amended version implemented in 2004 [22]. Both versions had a median value of 6 and we classified patients with a score of >6 on either version as having sustained a severe stroke. As the NIHSS score was not available for the full study period, we also used an alternative pragmatic classification for assessing severity which we termed “clinical severity”. We used three clinical indicators (Glasgow coma score, urinary continence, ability to swallow) which have been used previously as indicators of case severity at initial assessment in the acute phase [23]. We classified patients as having suffered a severe stroke if they met any of the following criteria on initial assessment – incontinent of urine, unable to swallow, Glasgow coma score <9, or if the patient died <2 days of stroke onset. We added the latter to take account of patients who had died of acute stroke before being admitted. Of the 1207 patients with severe stroke, 957 had urinary incontinence, 849 were unable to swallow, 241 had a Glasgow coma score <9, and 61 had died <2 days of stroke onset. Missing data frequencies were 60, 79, 38 and 0 respectively for the corresponding variables.

### Exposure to air pollution

We used modeled outdoor air pollution concentrations of particulate matter less than 10um in diameter (PM_10_) and nitrogen dioxide (NO_2_) that that had been produced for Greater London for 2002. The modeled concentrations were available at a very fine spatial scale (20 meter by 20 meter grid point resolution) and had been validated against measured concentration values (correlations: r = 0.90 for PM_10_; r = 0.91 for NO_2_) [24]. The model took into account a range of pollution sources and emissions including major and minor road networks modeled with detailed information on vehicle stock, traffic flows and speed for each road segment, pollution sources in the London Atmospheric Emissions Inventory including large and small regulated industrial processes, boiler plants, domestic and commercial combustion sources, agriculture, rail, ships and airports, and pollution carried into the area by prevailing winds. Within the study area, road traffic related pollution was the main contributor to spatial variation in pollution concentrations [24].

We used the 2001 UK census output area as the geographical unit of analysis. This was the smallest areal unit at which census population counts by five-year age band and sex were available. We calculated a population weighted average pollution concentration for each output area by taking the average of pollution concentrations assigned to all residential postcodes in an output area and weighting the average by the population count for each postcode. Postcode centroids had been assigned the pollution value of the nearest grid point. There was an average of five postcodes per output area.

### Statistical analysis

Differences in socioeconomic deprivation levels between areas may confound associations between air pollution and stroke. We therefore used the Income Domain of the Index of Multiple Deprivation from 2004, the main indicator of deprivation at the neighborhood level in England, as an indicator of socioeconomic deprivation at the area level [25]. The Index had been calculated for lower layer super-output areas (which typically comprise five output areas) and we assigned the value of the Income Domain to all output areas within each super-output area.

We modeled observed counts using Poisson regression in SAS with adjustment for any overdispersion. We calculated expected counts using indirect internal standardization to adjust for differences in age and sex between areas and entered the logarithm of these counts as the offset. Pollutants and deprivation were examined as continuous variables and as categorical variables grouped by tertile. Results are presented as rate ratios with 95% confidence intervals.

## Results

There were 948 census output areas in the study area with a total population of 267839, giving an average of 283 people per output area. The mean (SD) concentration of PM_10_ was 25.1 (1.2) ug/m^3^ with a range of 23.3 to 36.4 ug/m^3^. The mean (SD) concentration of NO_2_ was 41.4 (3.0) ug/m^3^ with a range of 35.4 to 68.0 ug/m^3^. The mean (SD) socioeconomic deprivation score was 0.23 (0.09) (range 0.01 to 0.46) with higher scores indicating higher levels of deprivation.

**Figure 1:**
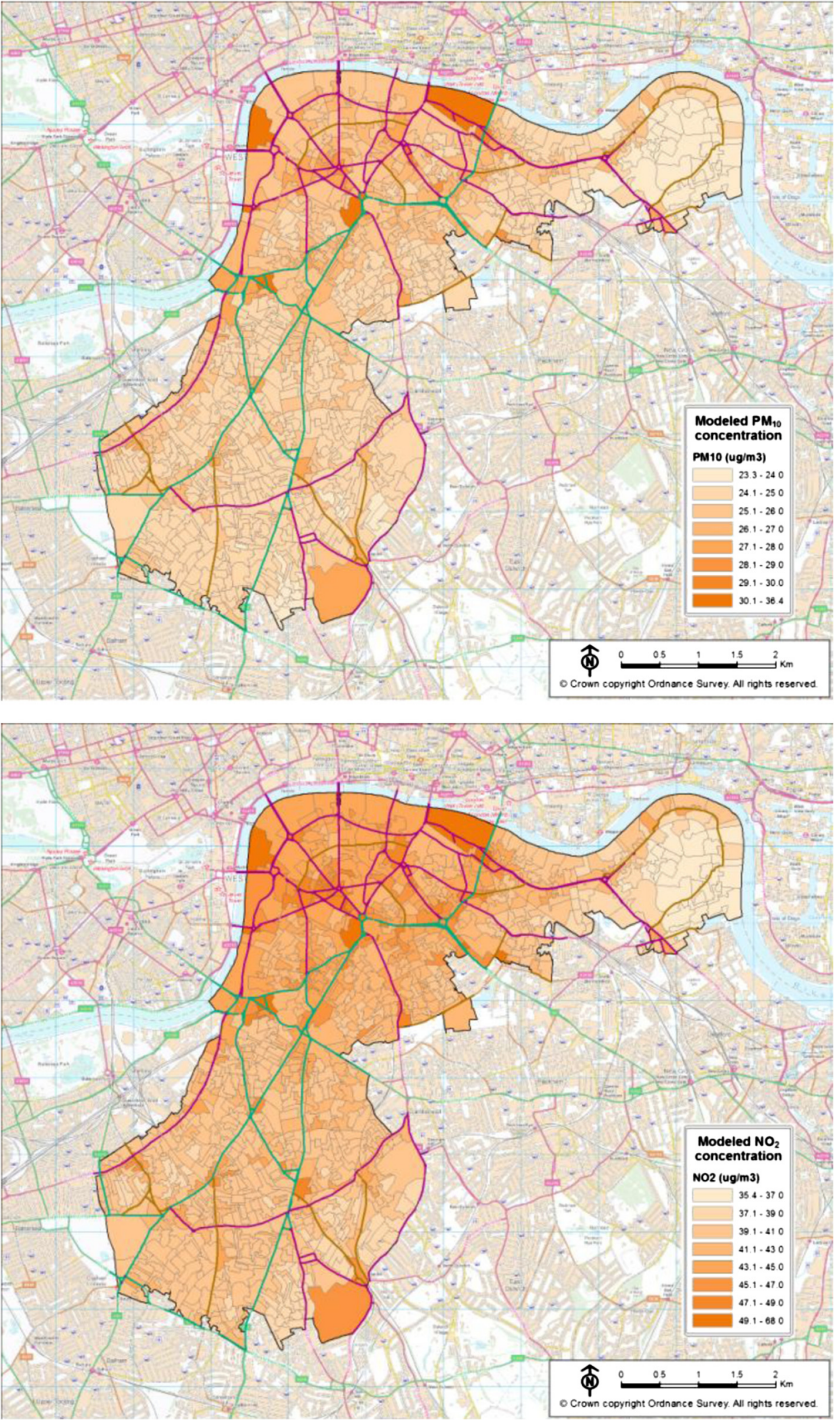
**Modeled PM**_**10 **_**and NO**_**2 **_**concentrations in census output areas in the South London Stroke Register study area, 2002.**

The concentration of air pollutants in output areas within the study area is shown in Figure 1. The study area was within the densely built urban environment of inner London. It can be seen that the concentrations of both PM_10_ and NO_2_ were related to the distribution of the road network, reflecting the fact that traffic related air pollution had a major influence on the spatial pattern of these pollutants within the study area.

There were 2492 incident cases of ischemic stroke occurring in 1995–2007 giving a crude annual incidence of 72 per 100,000 population. There were on average 2.63 (SD 2.15, range 0–14) ischemic stroke cases observed per census output area. With regard to observed counts for subtypes and severity, this average ranged from 0.14 (SD 0.37, range 0–2) for large artery atherosclerosis to 1.36 (SD 1.32, range 0–8) for mild stroke defined using the clinical severity classification. We mapped the stroke data and no unusual patterns or clustering were observed.

Of the 2492 ischemic strokes, 473 (19.0%) had TACI, 762 (30.6%) had PACI, 362 (14.5%) had POCI, 860 (34.5%) had LACI and the remaining 35 (1.4%) had no sub-classification assigned. Table 1 shows that patients with TACI had severe stroke on both NIHSS and clinical severity classifications (96.3% and 90.3% respectively). Patients with LACI tended to have less severe stroke (27.5% and 30.7% classified as having severe stroke based on NIHSS and clinical severity respectively).

**Table 1:** Characteristics of patients with incident ischemic stroke classified by subtype and severity in the South London Stroke Register study area, 1995-2007

**Classification**	**n**	**Mean (SD) age (years)**	**Male (%)**	NIHSS score >6 (%) [n]*	**Clinical severity - severe (%) [n]**
***Oxford classification***					
TACI	473	73.4 (13.5)	43.8	96.3	90.3
				[156/162]	[427/473]
PACI	762	72.3 (13.2)	48.0	54.2	46.6
				[187/345]	[355/762]
POCI	362	71.6 (12.4)	59.1	36.7	39.2
				[58/158]	[142/362]
LACI	860	70.9 (13.3)	51.3	27.5	30.7
				[102/371]	[264/860]
TOAST classification**					
Large artery atherosclerosis	136	70.9 (12.5)	54.4	57.0	46.3
				[61/107]	[63/136]
Cardioembolism	371	75.2 (13.5)	43.7	62.8	61.2
				[167/266]	[227/371]
Small vessel occlusion	376	69.6 (12.9)	54.3	26.9	24.2
				[79/294]	[91/376]
***NIHSS score***					
= < 6	541	69.4 (14.1)	55.6	-	17.2
					[93/541]
>6	510	73.0 (13.3)	47.7	-	71.6
					[365/510]
***Clinical severity***					
Mild	1285	68.9 (13.0)	56.3	24.5	-
				[145/593]	
Severe	1207	75.1 (12.7)	43.4	79.7	-
				[365/458]	

TACI - total anterior circulation infarct; PACI - partial anterior circulation infarct; POCI - posterior circulation infarct; LACI - lacunar infarct.

TOAST - Trial of Org 10172 in Acute Stroke Treatment etiological classification.

NIHSS - National Institutes of Health Stroke Scale.

*NIHSS from 2001; **TOAST from 2000.

There were 136 stroke cases classified as attributable to large artery atherosclerosis, 371 classified as attributable to cardioembolism and 376 classified as attributable to small vessel occlusion from 2000–2007 (Table 1). Patients with ischemic stroke caused by large artery atherosclerosis had more severe stroke (57.0% and 46.3% classified as having severe stroke based on NIHSS and clinical severity respectively). Similarly, patients with ischemic stroke caused by cardioembolism had more severe stroke (62.8% and 61.2% respectively). In contrast, patients classified as having ischemic stroke caused by small vessel occlusion had less severe stroke, with 26.9% and 24.2% classified as having severe stroke based on NIHSS and clinical severity respectively.

With regard to severity, 1051 patients with ischemic stroke were classified by severity based on the NIHSS score. All ischemic strokes could be classified by clinical severity, with 1207 (48.4%) classified as severe and 1285 (51.6%) classified as mild. Patients with severe stroke were older, with a lower proportion of males (Table 1). There was moderate concordance between clinical severity and NIHSS severity, assessed in the 1051 patients with severity classified using both measures (kappa statistic = 0.55, p < 0.001).

Table 2 gives rate ratios associated with increases in PM_10_ concentration. There were no significant associations between PM_10_ and any of the Oxford or TOAST subtypes or with NIHSS severity categories. There was, however, a significant association with mild stroke based on the clinical severity classification, when PM_10_ concentration was examined as a categorical variable by tertile. The rate ratio in the highest category of PM_10_ was 1.20 (1.05-1.38) relative to the lowest category (p = 0.009).

**Table 2:** **Ischemic stroke incidence rate ratios by severity and subtype associated with increases in outdoor PM**_10_** concentration in the South London Stroke Register study area, 1995-2007**

**Stroke category**	**n**	**Rate ratio (95% CI)**			
			**Category by tertile**		
		**Per IQR (1.1ug/m**^**3**^**) increase**	**Low**	**Intermediate**	**High**
***Oxford classification***					
TACI	473	1.03 (0.95-1.13)	1	1.02 (0.81-1.27)	1.07 (0.85-1.34)
PACI	762	1.00 (0.93-1.07)	1	0.99 (0.83-1.19)	1.06 (0.89-1.26)
POCI	362	1.00 (0.90-1.11)	1	1.05 (0.81-1.36)	1.11 (0.86-1.44)
LACI	860	0.96 (0.89-1.03)	1	0.95 (0.80-1.12)	1.00 (0.85-1.18)
TOAST classification*					
Large artery atherosclerosis	136	1.05 (0.89-1.23)	1	0.90 (0.59-1.37)	1.05 (0.70-1.59)
Cardioembolism	371	1.03 (0.93-1.13)	1	1.00 (0.77-1.30)	1.15 (0.89-1.48)
Small vessel occlusion	376	0.93 (0.83-1.04)	1	1.21 (0.95-1.56)	1.04 (0.80-1.35)
NIHSS severity**					
NIHSS = <6	541	0.95 (0.87-1.04)	1	1.20 (0.97-1.48)	1.09 (0.88-1.35)
NIHSS >6	510	1.03 (0.95-1.12)	1	1.06 (0.86-1.32)	1.08 (0.87-1.35)
***Clinical severity***					
Mild	1285	1.04 (0.99-1.09)	1	1.06 (0.92-1.22)	1.20 (1.05-1.38)
Severe	1207	0.95 (0.89-1.01)	1	0.95 (0.82-1.09)	0.92 (0.80-1.06)

TACI - total anterior circulation infarct; PACI - partial anterior circulation infarct; POCI - posterior circulation infarct; LACI - lacunar infarct.

TOAST - Trial of Org 10172 in Acute Stroke Treatment etiological classification.

NIHSS - National Institutes of Health Stroke Scale.

*TOAST from 2000; **NIHSS from 2001.

Table 3 gives rate ratios associated with increases in NO_2_ concentration and the overall picture was very similar to that for PM_10_. There were no significant associations with any of the Oxford or TOAST subtypes or with either of the NIHSS severity categories. However, there was a significant association between NO_2_ concentration examined using tertiles and mild stroke. The rate ratio in the highest NO_2_ category was 1.22 (1.06-1.40) compared with the lowest NO_2_ category (p = 0.005).

**Table 3:** **Ischemic stroke incidence rate ratios by severity and subtype associated with increases in outdoor NO**_2_** concentration in the South London Stroke Register study area, 1995-2007**

**Stroke category**	**n**	**Rate ratio (95% CI)**			
			**Category by tertile**		
		**Per IQR (3.2ug/m**^**3**^**) increase**	**Low**	**Intermediate**	**High**
***Oxford classification***					
TACI	473	1.03 (0.93-1.14)	1	1.10 (0.88-1.38)	1.06 (0.84-1.34)
PACI	762	1.01 (0.93-1.09)	1	1.04 (0.87-1.24)	1.07 (0.90-1.28)
POCI	362	1.01 (0.90-1.13)	1	1.07 (0.82-1.39)	1.07 (0.83-1.39)
LACI	860	0.98 (0.91-1.06)	1	1.06 (0.89-1.25)	1.07 (0.91-1.27)
TOAST classification*					
Large artery atherosclerosis	136	1.07 (0.90-1.28)	1	0.96 (0.63-1.46)	1.03 (0.68-1.56)
Cardioembolism	371	1.05 (0.94-1.17)	1	1.08 (0.83-1.40)	1.16 (0.90-1.50)
Small vessel occlusion	376	0.95 (0.84-1.07)	1	1.21 (0.94-1.56)	1.09 (0.84-1.42)
NIHSS severity**					
NIHSS = <6	541	0.96 (0.87-1.06)	1	1.12 (0.91-1.38)	1.06 (0.86-1.32)
NIHSS >6	510	1.05 (0.95-1.15)	1	1.07 (0.86-1.34)	1.14 (0.91-1.42)
***Clinical severity***					
Mild	1285	1.06 (1.00-1.12)	1	1.10 (0.96-1.26)	1.22 (1.06-1.40)
Severe	1207	0.95 (0.89-1.01)	1	1.04 (0.91-1.20)	0.94 (0.81-1.09)

TACI - total anterior circulation infarct; PACI - partial anterior circulation infarct; POCI - posterior circulation infarct; LACI - lacunar infarct.

TOAST - Trial of Org 10172 in Acute Stroke Treatment etiological classification.

NIHSS - National Institutes of Health Stroke Scale.

*TOAST from 2000; **NIHSS from 2001.

## Discussion

### Summary of results

We found no evidence of association between outdoor PM_10_ and NO_2_ concentrations, both indicators of road traffic related pollution in our study area, and the incidence of ischemic stroke subtypes classified using either the Oxford clinical classification or the TOAST aetiological classification. In addition, we found no significant associations between these pollutants and stroke severity using NIHSS severity categories. However, we found that outdoor concentrations of both PM_10_ and NO_2_ appeared to be associated with increased incidence of mild but not severe ischemic stroke, classified using the pragmatic clinical severity classification.

### Comparison with previous studies

One previous study has found that exposure to ultrafine particles and NO_x_ were both associated with an increased risk of mild but not severe stroke, consistent with our results [14]. The authors analyzed daily time series data on air pollutants and stroke admissions and the focus of their study was on acute effects as daily time series studies by the nature of their design only capture short term effects. In contrast, our ecological study design would have captured both acute and chronic effects of air pollution exposure on stroke risk.

Andersen et al. used severity as a proxy to differentiate between small and large vessel occlusion and hypothesized that air pollution is more strongly linked to small vessel cerebrovascular disease [14]. Air pollution may cause ischemic stroke through various mechanisms including systemic inflammation and activated thrombosis pathways, arteriolar narrowing and impaired vasodilatation, and accelerated progression of atherosclerosis [26]. Lacunar strokes are a clinical manifestation of cerebrovascular small vessel disease and potential mechanisms include microatheroma of deep perforating arteries, vascular endothelial dysfunction and genetic predisposition to lacunar stroke [27]. The putative mechanisms by which air pollution could cause ischemic stroke are consistent with potential mechanisms mediating the link between cerebrovascular small vessel disease and ischemic stroke.

We however found no significant associations between air pollutants and small vessel disease stroke. In addition, we found no significant associations with lacunar stroke and our results do not appear to be consistent with the air pollution and small vessel disease hypothesis. One possible explanation is that we had a relatively small number of cases especially for small vessel disease, resulting in inadequate power to detect associations. Misclassification of etiological and clinical subtypes could also have occurred, resulting in bias towards a null association. In contrast to our results, three previous studies found associations between outdoor air pollutants and stroke due to small vessel disease or lacunar stroke [7-9]. However, a further study found no association with small vessel disease stroke [6]. Three of the above studies also found associations with stroke due to large vessel disease [6-8], suggesting that any adverse air pollution effect is not specific to small vessel disease. It is worth noting though that all four studies found no association with cardioembolic stroke [6-9], consistent with our results.

There is a potential explanation, speculative at this stage, which might explain the stronger association between air pollution and mild stroke, and which does not require the small vessel disease hypothesis. It may be that thrombus formation triggered by air pollutants is smaller, less dense or more easily broken down. In addition, atheromatous plaques induced by air pollutants may be more modest in size. With this hypothesis, the air pollution effect would not need to be preferentially associated with small vessel disease stroke as ischemic stroke caused by air pollutants could affect large and small arteries alike but the resulting clinical picture would tend to be that of a mild stroke.

We previously observed that air pollution exposure was more strongly associated with stroke mortality than with stroke hospital admissions [13]. Others have also reported a stronger association with fatal than with non-fatal stroke [28]. This suggests that air pollution ought to be more strongly associated with severe stroke and is therefore not consistent with the results we have observed. However, a possible alternative explanation is that whilst air pollution is more strongly associated with mild stroke, exposure to high pollution reduces survival after stroke and it is the latter which accounts for the stronger association seen with stroke mortality. In this regard, we have previously reported a strong independent adverse effect of outdoor air pollution exposure on survival after stroke [24].

### Internationally significant novelty

The internationally significant novelty of our study results from two aspects. Firstly, air pollution is a widespread environmental hazard, with increasing levels of road traffic related air pollution encountered in many high and middle income countries across the globe. Secondly, ours is the first study to examine the chronic effects of outdoor air pollution on the incidence of ischemic stroke subtypes.

### Limitations

There are a number of potential limitations to our study. The ecological study design is susceptible to ecological bias, which is the situation where the association seen at the area level is different from that which exists at the individual level. Ecological bias cannot be ruled out in our study. However, we used a small area level ecological study design which would have mitigated ecological bias as small areas tend to be relatively more homogenous in terms of population characteristics and exposure to environmental pollutants [10]. In addition, we compared effects on different stroke subtypes and severity within the same ecological study and any bias inherent in the study design might be expected to have similar effects on the different severity and subtype groups examined. We used modeled exposure from a validated model [24]. However, the modeled estimates were for a single year and we did not have data to take account of general population mobility over the study period. In addition, we did not have data to take into account daily population movements. Both these limitations are likely to have resulted in some exposure misclassification. The relatively narrow distribution of air pollution values within the study area may also have contributed to the general lack of associations seen.

The stroke register was estimated to have missed 12-20% of cases and there may have been errors in denominator population estimates, leading to further error in our effect estimates [17,18,29]. The incomplete case capture is also likely to explain the lower ischemic stroke incidence we observed compared with other studies [30]. We adjusted for deprivation using the Income Domain of the Index of Multiple Deprivation but the possibility of residual confounding exists as deprivation may not have been fully adjusted for using this indicator. Although the stroke register contained information on other potential confounders such as smoking, we did not have equivalent information for the denominator population at the census output area level which would have allowed adjustment for these potential confounders.

We used a non-standard clinical severity classification system for pragmatic reasons and although this had the advantage of being able to classify all ischemic strokes as mild or severe, there is likely to have been some misclassification. This is indicated by the kappa statistic which showed only moderate concordance between the clinical severity and NIHSS severity classifications. Whilst we found significant associations between pollutants and mild stroke, we found no significant associations when using the Oxford and TOAST classifications. Potential explanations include inadequate power due to the smaller numbers of cases available for these analyses and misclassification of subtypes. The associations we found may have arisen by chance as we have carried out a number of comparisons, despite the small p-values and consistency with a previous study [14]. Our findings therefore need to be interpreted with caution given the potential limitations.

## Conclusions

In summary, we found no evidence of association between outdoor PM_10_ and NO_2_ concentrations and ischemic stroke subtypes but there was a suggestion that living in areas with elevated outdoor PM_10_ and NO_2_ concentrations might be associated with increased incidence of mild, but not severe, ischemic stroke. Further studies are needed to investigate the links between air pollutants, severity and subtypes of stroke.

## Competing interests

The authors declare that they have no competing interests.

## Authors’ contributions

RM conceived the idea for this study. CDW had established the South London Stroke Register. SDB carried out the air pollution modeling. TP linked the data. RM and TP carried out the analysis with advice from MJC. RM wrote the first draft of the manuscript. All authors read, made substantial contributions to and approved the manuscript.

## Authors’ information

Ravi Maheswaran MD, Reader in Epidemiology and Public Health. Tim Pearson MSc, Research Associate in Public Health. Sean D Beevers PhD, Principal Air Quality Scientist. Michael J Campbell PhD, Professor of Medical Statistics. Charles D Wolfe MD, Professor of Public Health Medicine.
